# Supplementary material for: Exploring the Effects of Various Two-Dimensional Supporting Materials on the Water Electrolysis of Co-Mo Sulfide/Oxide Heterostructure
Source: Nanomaterials (Basel). 2023 Aug 31;13(17):2463. doi: 10.3390/nano13172463 (PMC10490037; doi:10.3390/nano13172463)
Supplement: Supplementary file 1 [file nanomaterials-13-02463-s001.zip › nanomaterials-2582549-supplementary.pdf]

Supporting information

Exploring the Effects of Various Two-Dimensional Supporting Materials on the Water Electrolysis of Co-Mo Sulfide/Oxide Heterostructure

Ngoc-Diem Huynh, Won Mook Choi * and Seung Hyun Hur *

School of Chemical Engineering, University of Ulsan, Daehak-ro 93, Nam-gu, Ulsan 44610, Republic of Korea; diemhuynh0908@gmail.com

* Correspondence: wmchoi98@ulsan.ac.kr (W.M.C.); shhur@ulsan.ac.kr (S.H.H.)

Experimental

Chemicals

Cobalt acetate ($Co(CH_3COO)_2.4H_2O$), glycerol ($C_3H_8O_3$), isopropanol (C_3H_8O), ethanol (C_2H_6O), and hydrazine monohydrate ($N_2H_4.H_2O$) were purchased from Daejung chemicals. Thioacetamide (TAA) (C_2H_5NS), ammonium molybdate tetrahydrate ($(NH_4)_6Mo_7O_{24}.4H_2O$), melamine ($C_3H_6N_6$), Nafion 5%, ruthenium oxide (RuO_2), Pt/C 20%, and potassium hydroxide (KOH) were bought from Sigma-Aldrich. All of the chemicals were used without any purification after purchasing. Deionized water (DI) was used for all reactions.

Synthesis

Co-glycerate precursor: In a typical synthesis procedure, 0.104 g of $Co(CH_3COO)_2.4H_2O$ and 8 ml of glycerol were dissolved into 40 mL of C_3H_8O to form a transparent dark-green solution. The solution was then transferred to a Teflon-lined stainless-steel autoclave and kept at 180 °C for 6 h. After cooling to room temperature naturally, the purple precipitate was separated by filtration, washed several times with ethanol, and dried in an oven at 70 °C.

Material characterization

A DXR Raman microscope (Thermo Scientific) was employed to obtain the Raman spectra for all samples; the range collected was from 100 to 1700 cm-1, with a 532 nm monochromatic excitation source. The X-ray diffraction (XRD) patterns were obtained by an A Rigaku X-ray diffractometer (D/MAZX 2500V/PC, Japan) with Cu Kα radiation (0.154 nm) and a scan range of 10-90°. The X-ray photoelectron spectroscopy (XPS) was performed on thermo ESCALAB 250 Xi, Thermo Fisher Scientific, USA, with Al Kα X-ray radiation (1486.6 eV). The morphology of materials was observed using field emission scanning electron microscopy (FESEM, Hitachi High-Tech Corporation, SU7000, Japan). The degas and nitrogen adsorption–desorption revealed the surface area and pore size of the samples, which were obtained using a Quantachrome Quadrasorb SI automated surface area and pore size analyzer.

Electrochemical measurement

All electrochemical experiments were performed in an alkaline medium (1 M KOH) using a three-electrode system (BioLogic, Science Instruments) at room temperature. The system included a reference electrode (RE), a counter electrode (CE), and a working electrode (WE). In the system, the RE was a Hg/HgO electrode, the CE was a graphite rod, and carbon paper (CC) was the WE which was washed with ethanol and DI before use. To prepare the WE, 3mg of catalyst was added in 490 μL of ethanol and 10 μL of Nafion 5%, and then this mixture was sonicated for 30 minutes to obtain the homogeneous solution. Finally, 200 μL of prepared solution was dropped on CC (1x1 cm^2), and the mass loading catalyst on CC was 1.2 mg/ cm^2. The RE was calibrated with respect to the reversible hydrogen electrode (RHE) in the system of Pt as WE and 1 M KOH saturated hydrogen. The electrode potential of Hg/HgO was 0.92 V at a current of 0 mA, as shown in **Fig. S11**. As a result, the formula to convert the potential of RE (Hg/HgO) to RHE was given by $E_{RHE}=E_{Hg/HgO}+0.92$ (V). The linear sweep voltammetry (LSV) was carried out at 5 mV/s in the potential range of 1-1.72 V versus RHE. The electrochemical impedance spectroscopy (EIS) was obtained over a frequency range of 100 kHz to 0.1 Hz at 1.50 V vs RHE. The interface charge-transfer resistance (R_{ct}) is equal to the diameter of the semicircle in the Nyquist plot. The cyclic voltammetry (CV) was run at 20, 40, 60, 80, and 100 mV/s in the range of 1.07-1.17 V to acquire the electrochemical double layer capacitance (C_{dl}), which was the linear slope of the $\Delta j = (j_a - j_c)/2$ at 1.12 V versus different scan rates. The electrochemical surface area (ECSA) of catalysts was determined using ECSA = C_{dl}/C_s, where the specific capacitance C_s was 40 μF/cm^2 [1]. Chronoamperometry (CA) and Chronopotentiometry (CP) were conducted at 10 mA/cm^2.

Figures and Tables

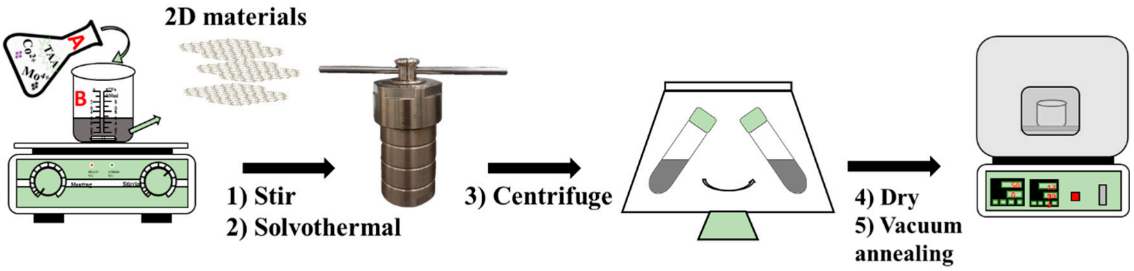

Scheme S1: The synthesis procedure of CMSO@2D

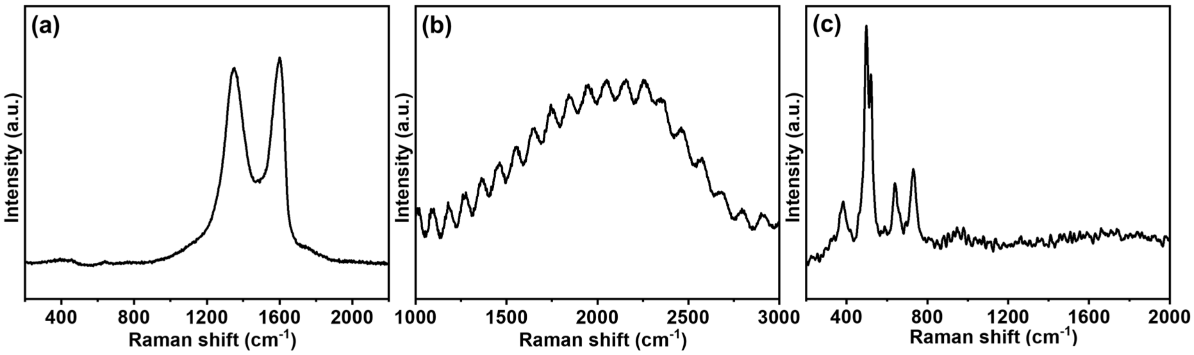

Fig. S1: Raman spectrum of (a) rGO, (b) gC_3N_4, and (c) SiSh

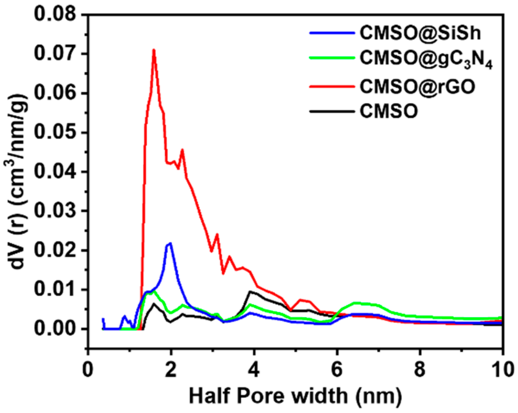

Fig. S2: Pore size distribution by density functional theory method of various materials

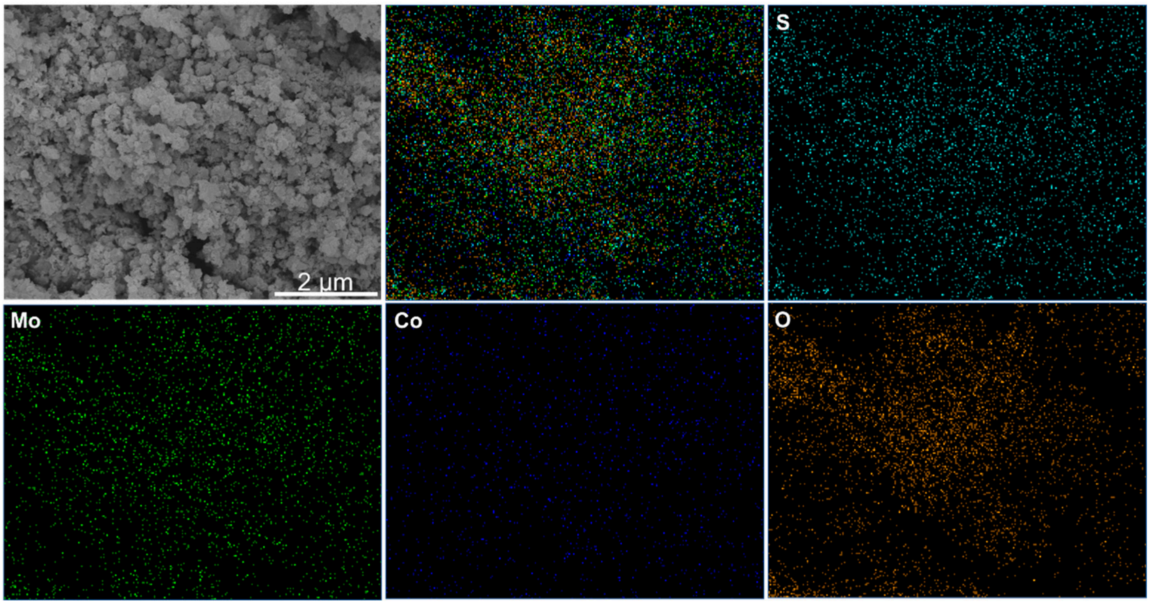

Fig. S3: Elemental mapping of CMSO

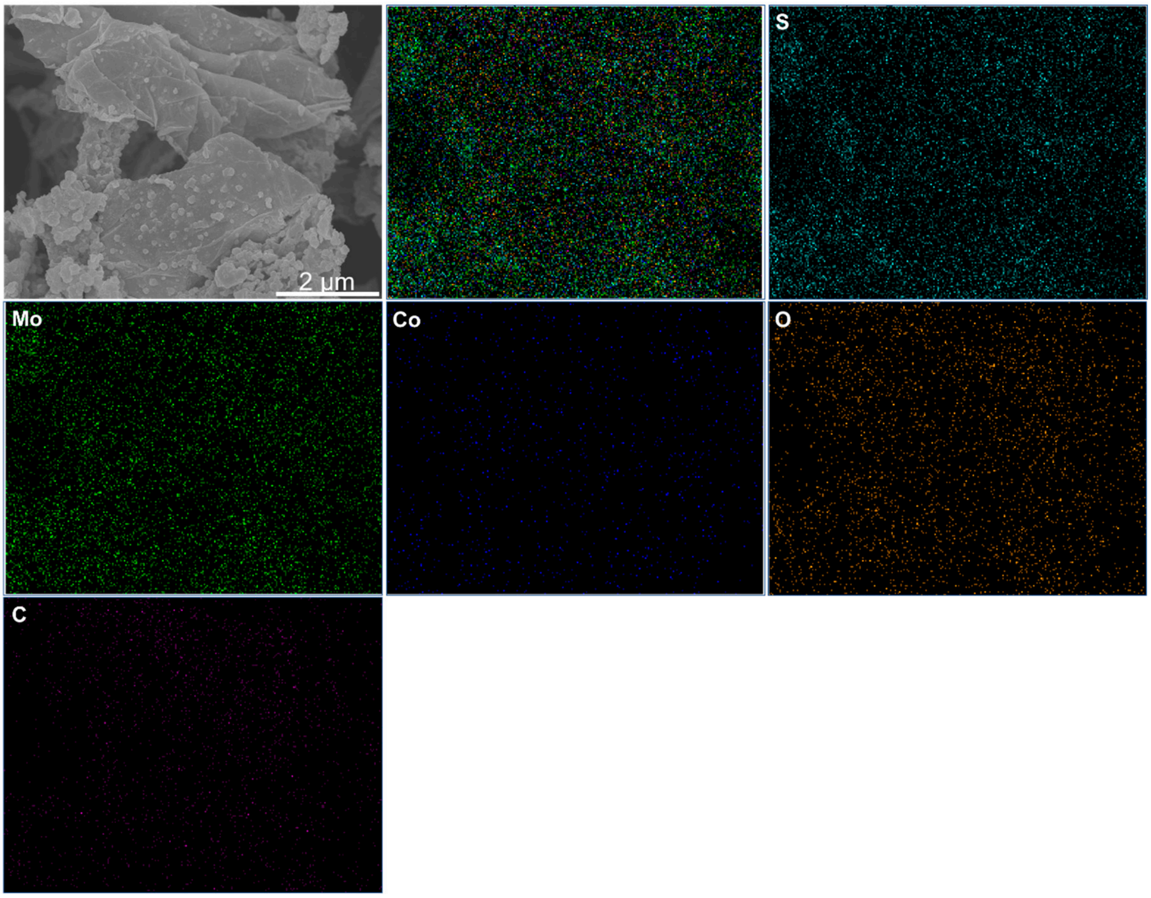

Fig. S4: Elemental mapping of CMSO@rGO

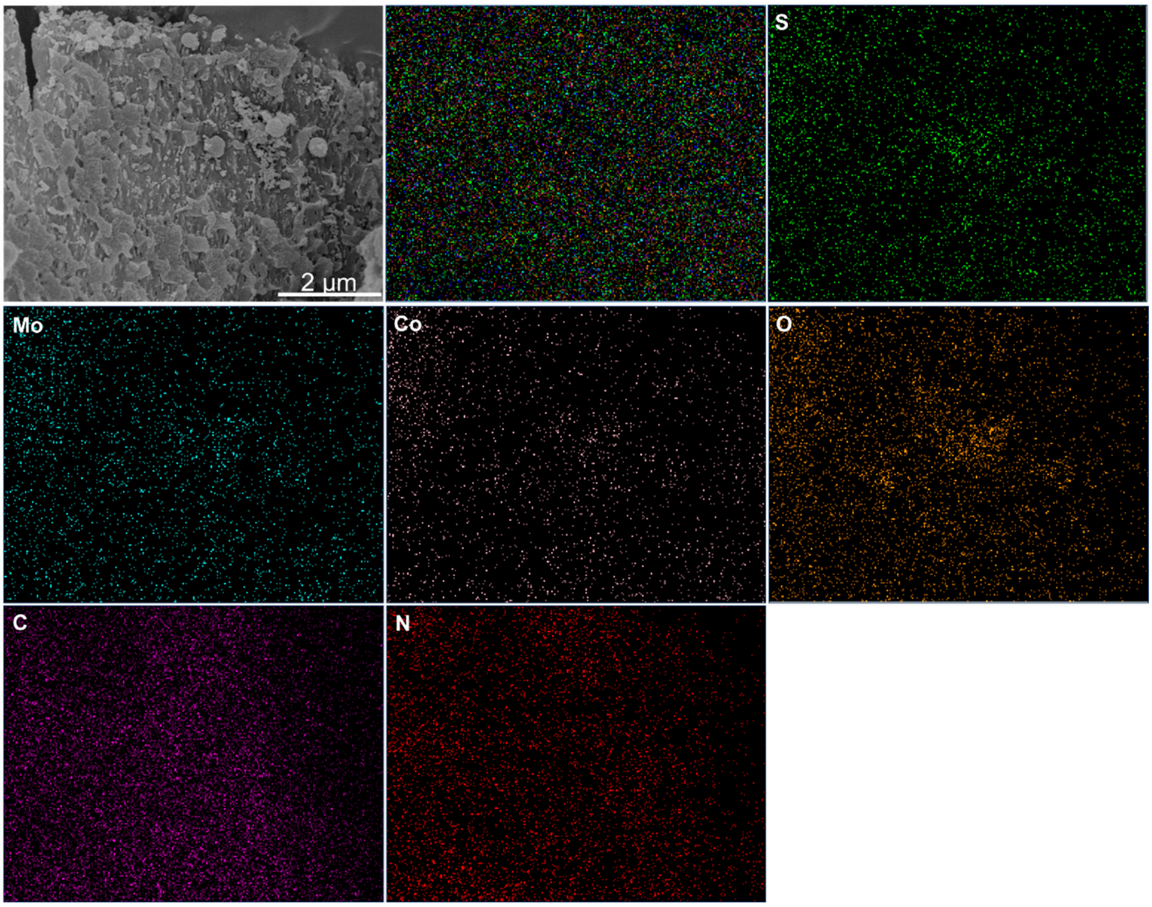

Fig. S5: Elemental mapping of $CMSO@gC_3N_4$

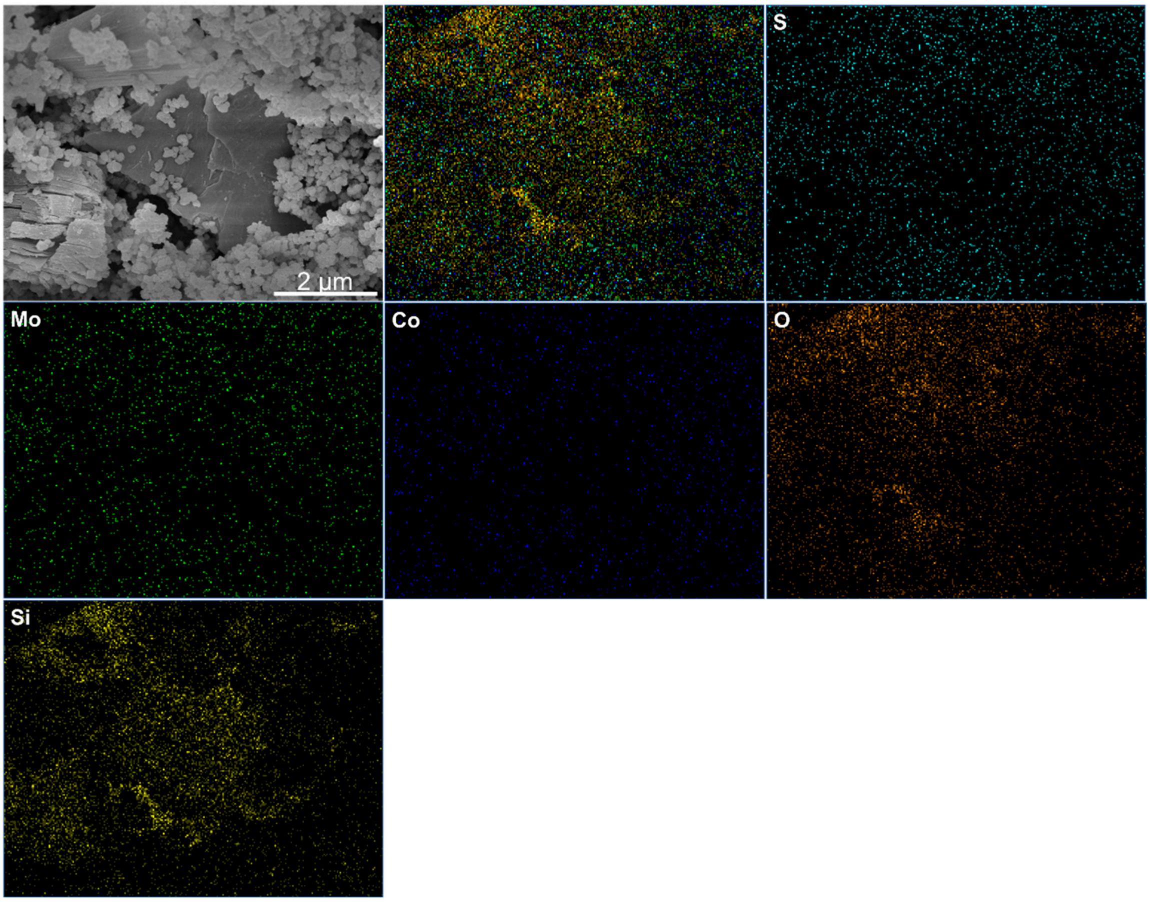

Fig. S6: Elemental mapping of CMSO@SiSh

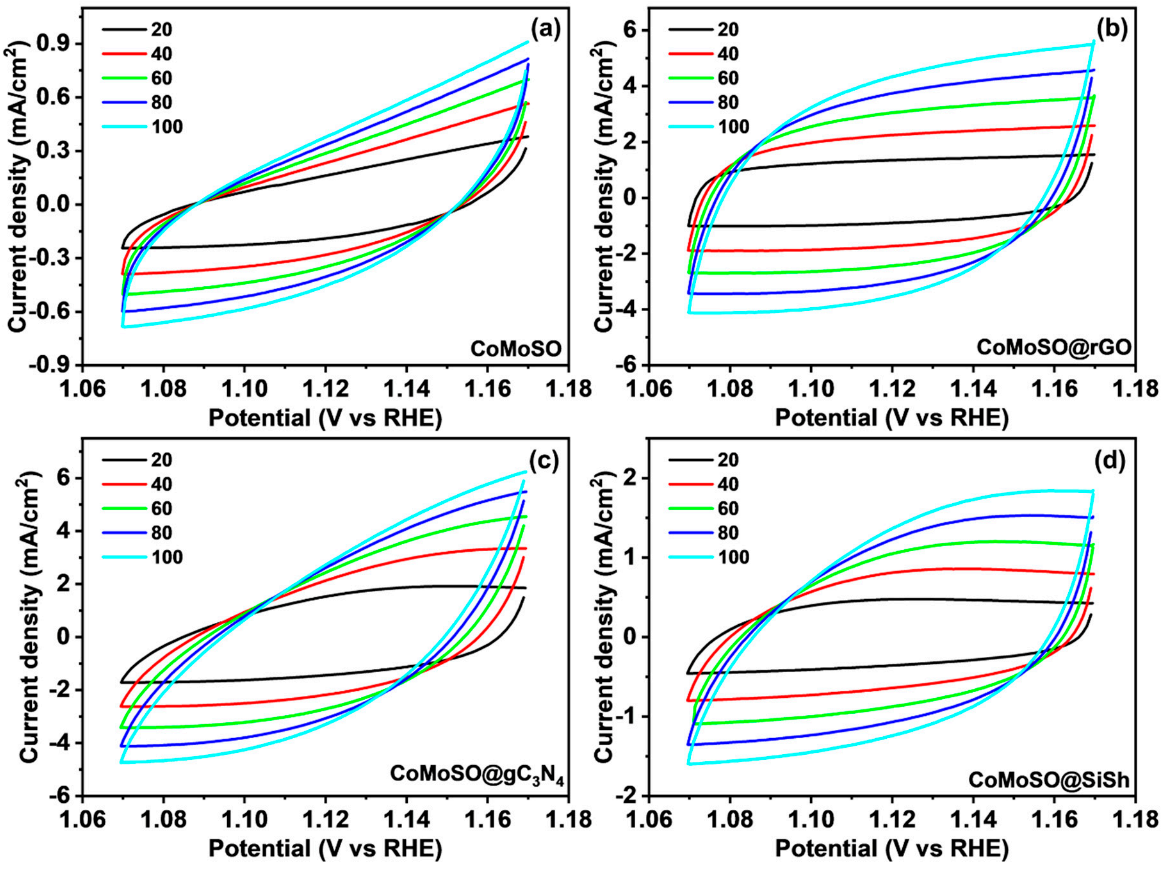

Fig. S7: Cyclic voltammetry curves at scan rates from 20 to 100 mV/s of prepared materials

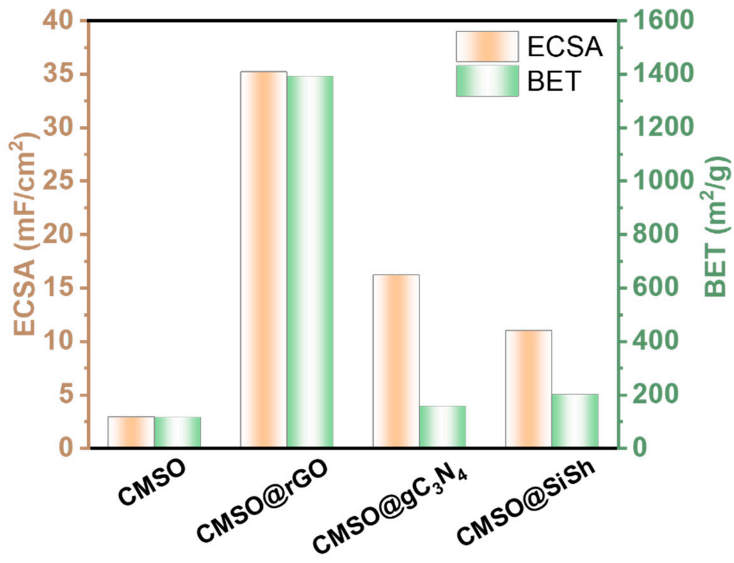

Fig. S8: Comparison of BET and ECSA of synthesized materials

Table S1: Comparison of OER activity in 1 M KOH of different electrocatalysts

Materials	Overpotential At 10 mA/cm^2 (mV)	Tafel slope (mA/dec)	Stability (hour)	Reference
CoS_2-MoS_2 hollow spheres	288	62.1	10	[2]
CoS_2–MoS_2 nanosheet	266	-	24	[3]
Cobalt Covalent Doping in MoS_2	260	85	11	[4]
Co-Mo-S-2/NF	294	65.85	120	[5]
MoS_2–CoS_2@PCMT	215	93	20	[6]
N-CoS_2/Graphene	260	56.3	8	[7]
MoS_2/$NiCo_2O_4$	322	113	12	[8]
CMSO@rGO	259	85	40	**This work**

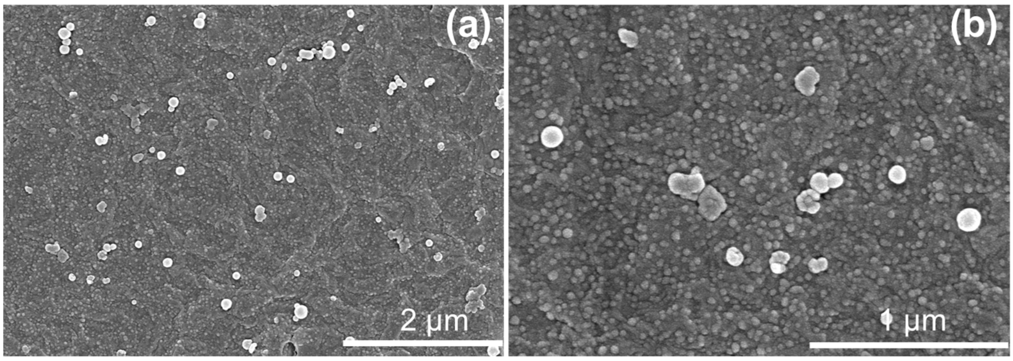

Fig. S9: FESEM of CMSO@rGO at different magnifications after OER

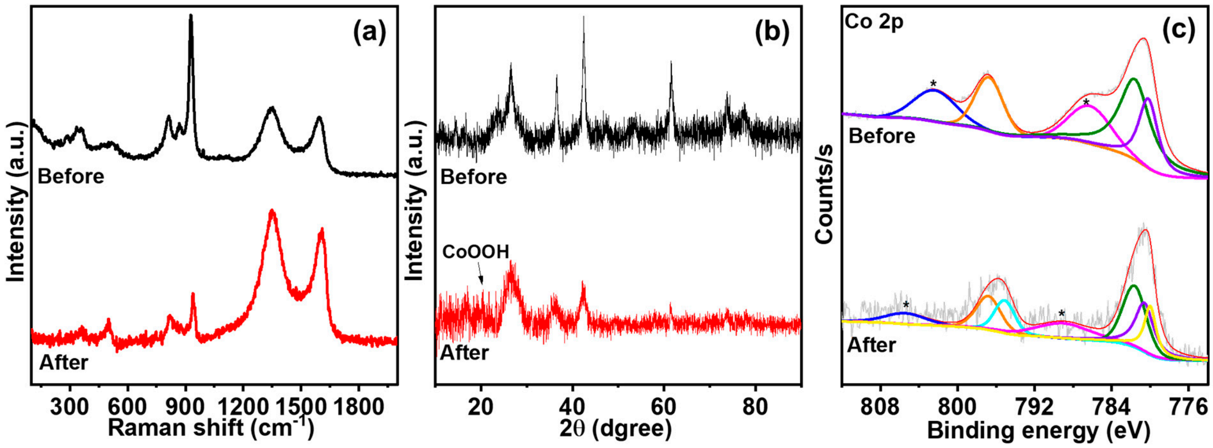

Fig. S10: Comparison of (a) Raman spectrum, (b) XRD pattern, and (c) XPS of Co 2p of CMSO@rGO before and after OER

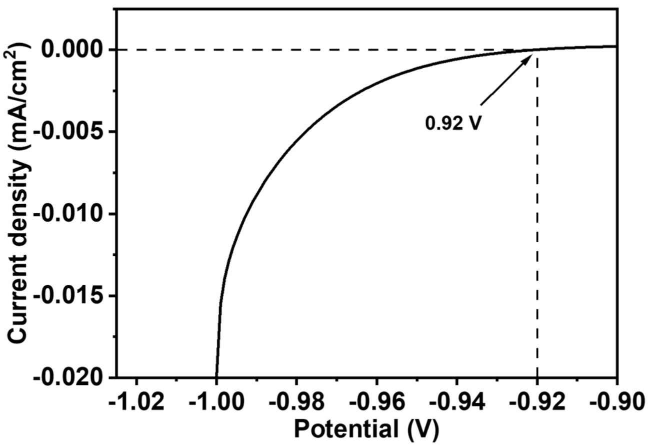

Fig. S11: Calibration of Hg/HgO electrode

References

1. McCrory, C. C. L.; Jung, S.; Peters, J. C.; Jaramillo, T. F., Benchmarking Heterogeneous Electrocatalysts for the Oxygen Evolution Reaction. *Journal of the American Chemical Society* **2013,** *135* (45), 16977-16987.
2. Ganesan, V.; Kim, J., Multi-shelled CoS2–MoS2 hollow spheres as efficient bifunctional electrocatalysts for overall water splitting. *International Journal of Hydrogen Energy* **2020,** *45* (24), 13290-13299.
3. Li, Y.; Wang, W.; Huang, B.; Mao, Z.; Wang, R.; He, B.; Gong, Y.; Wang, H., Abundant heterointerfaces in MOF-derived hollow CoS2–MoS2 nanosheet array electrocatalysts for overall water splitting. *Journal of Energy Chemistry* **2021,** *57*, 99-108.
4. Xiong, Q.; Wang, Y.; Liu, P.-F.; Zheng, L.-R.; Wang, G.; Yang, H.-G.; Wong, P.-K.; Zhang, H.; Zhao, H., Cobalt Covalent Doping in MoS2 to Induce Bifunctionality of Overall Water Splitting. *Advanced Materials* **2018,** *30* (29), 1801450.
5. Zhao, Z.-Y.; Li, F.-L.; Shao, Q.; Huang, X.; Lang, J.-P., Co-Modified MoS2 Hybrids as Superior Bifunctional Electrocatalysts for Water Splitting Reactions: Integrating Multiple Active Components in One. *Advanced Materials Interfaces* **2019,** *6* (11), 1900372.
6. Yang, J.; Chai, C.; Jiang, C.; Liu, L.; Xi, J., MoS2–CoS2 heteronanosheet arrays coated on porous carbon microtube textile for overall water splitting. *Journal of Power Sources* **2021,** *514*, 230580.
7. Tong, Y.; Sun, Q.; Chen, P.; Chen, L.; Fei, Z.; Dyson, P. J., Nitrogen-Incorporated Cobalt Sulfide/Graphene Hybrid Catalysts for Overall Water Splitting. *ChemSusChem* **2020,** *13* (18), 5112-5118.
8. Tao, B.; Yang, L.; Miao, F.; Zang, Y.; Chu, P. K., An MoS2/NiCo2O4 composite supported on Ni foam as a bifunctional electrocatalyst for efficient overall water splitting. *Journal of Physics and Chemistry of Solids* **2021,** *150*, 109842.
